# Influence of the Deqi Sensation by Suspended Moxibustion Stimulation in Lumbar Disc Herniation: Study for a Multicenter Prospective Two Arms Cohort Study

**DOI:** 10.1155/2013/718593

**Published:** 2013-07-17

**Authors:** Rixin Chen, Mingren Chen, Jun Xiong, Tongsheng Su, Meiqi Zhou, Jianhua Sun, Zhenhai Chi, Bo Zhang, Dingyi Xie

**Affiliations:** ^1^The Affiliated Hospital with Jiangxi University of TCM, No. 445 Bayi Avenue, Nanchang, China; ^2^Shanxi TCM Hospital, Xi'an, China; ^3^The First Affiliated Hospital with Anhui University of TCM, Hefei, China; ^4^Jiangsu TCM Hospital, Nanjing, China

## Abstract

Moxibustion stimulates the Deqi (Qi arrival) phenomenon. Many clinical observations have documented that the character of the Deqi was a composite heat-sensitive moxibustion sensation. In this prospective multicentre comparative observational nonrandomized study, 92 patients with moderate to severe LDH were included. This study consisted of two parallel arms (A: heat-sensitive moxibustion sensation group; B: nonheat-sensitive moxibustion sensation group). Moxibustion was applied in the following three acupuncture points simultaneously: Da Changshu (BL25), Wei Zhong (BL40), and A-Shi acupuncture point (tenderness). The adjusted mean total Modified-JOA score showed significant differences between the groups in the first week (10.32 ± 4.27 95% CI [9.23 ~ 11.40] versus control group 12.42 ± 5.02 [11.62 ~ 13.69], *P* = 0.03). The outcome in the second week also presented significant differences in both groups (7.62 ± 4.80 [6.46 ~ 8.77] versus 10.56 ± 4.75 [9.35 ~ 11.76], *P* = 0.005). Significant differences were also manifested in the follow-up period (*P* = 0.007). It can be inferred that the existence of the Deqi (heat-sensitive moxibustion sensation) phenomenon in the process of suspended moxibustion is closely related to the curative effect, and arrival of heat-sensitive moxibustion sensation could improve the clinical curative effect of moxibustion.

## 1. Background

Lumbar disc herniation (LDH) is one of the major chronic musculoskeletal diseases that are highly prevalent. In China, about 10%~15% patients with low back pain were diagnosed with LDH [1]. It seriously affects patient's quality of life (QoL) and leads to economic burden [2, 3]. Epidemiological studies from several countries showed that the prevalence of LDH frequently appeared in adults at the age of 30~55 [4]. LDH causes symptoms of sciatica and possible foot pain, numbness, or weakness. In most of the cases, a conservative attitude with different types of physiotherapy is preferred as the first choice [5, 6]. Most of patients with low back pain responded well to conservative therapy [7]. Absolute indications for surgery include altered bladder function and progressive muscle weakness, but these are rare. Therefore, many studies have already reported encouraging results in the treatment of LDH by acupuncture and moxibustion [8].

Moxibustion is a traditional Chinese method of treatment, which applies the heat generated by burning moxa (it is also called Mugwort or Moxa) to stimulate on the acupuncture points. Suspended moxibustion is commonly used, and it refers to application of the burning moxa stick on the acupuncture points at a distance. The results of a recent meta-analysis of six randomized controlled trials (RCTs) on moxibustion for LDH manifested that moxibustion presented a favorable effect on LDH symptom scores compared with that of the drug [RR = 1.91, 95%  CI  (1.01, 3.60)] [9].

According to traditional Chinese medicine (TCM), the Deqi is the key point to the clinical efficacy of acupuncture and moxibustion [10]. The Deqi is a term originated from *Huangdi Neijing,* also known as “Qi arrival.” In the part of *Miraculous pivot, the chapter of nine needles and twelve sources* said: “The key point of acupuncture is the arrival of Qi, it ensures therapeutic effect. It resembles the wind over blows the cloud, soon the sky is clear.” [11]. The Deqi's primary connotation is the endogenous Qi of regulation stimulated by acupuncture and moxibustion, which is closely related to the curative effect [12]. When Qi arrives at one part of the body, it can treat the diseases nearby.

Numerous studies have now shown that moxibustion stimulation stimulated a unique Deqi, heat-sensitive moxibustion sensation [13, 14]. In the process of moxibustion treatment, the researchers discovered that, when human body is in morbid condition, related acupuncture points were quite sensitive to moxa's heat and produced nonlocal or nonsuperficial heat sensation, such as penetrating heat, expanding heat, and transmitting heat [15]. This phenomenon resembles the one that occurs when the Deqi appears after moxibustion rather than local heat sensation and surface glow of the skin. The researchers named the phenomenon as heat-sensitive phenomenon of moxibustion or acupuncture point's heat-sensitive phenomenon, and it belongs to the Deqi phenomenon of moxibustion therapy [16].

However, there is lack of experimental data to indicate the difference of heat-sensitive moxibustion sensation (the Deqi stimulated by moxibustion) compared with conventional local superficial heat sensation (non-Deqi by moxibustion stimulation). For LDH especially, is it necessary for moxibustion to produce the phenomenon of obtaining Qi in order to improve the curative effect? Therefore, it would be valuable to know whether there is difference between the moxibustion sensations in the treatment of LDH. Therefore, we planned the rigorous multi centre prospective cohort study trial to investigate the difference.

## 2. **Methods**

### 2.1. Objective

The aim of this study is to determine the effectiveness of heat-sensitive moxibustion sensation and non-heat-sensitive moxibustion sensation in the treatment of patients with moderate to severe LDH in China.

### 2.2. Sample Size

The effective rate was used to determine sample size. There are few reports in the literature of clinical trials of control mode for LDH. Based on our earlier randomized controlled pretrial in the Affiliated Hospital of Jiangxi University of TCM, we believe that the effective rate for LDH is approximately 45% when adopting the non-heat-sensitive moxibustion sensation and should be increased to 75% when using the heat-sensitive moxibustion sensation. Based on 90% power at *P* = 0.05, 38 participants were included in each group to be calculated with the SPSS 13.0 programme. 20% loss was allowed to follow up a total of 46 participants were included in each group, with 92 participants in total.

Moreover,

(1) n={Z1−a2pq+Z1−βp1(1−p1)+p2(1−p2)p1−p2}2,p=(p1+p2)2 q=1−p.

### 2.3. Design

The patients were referred by the doctors and acupuncturists from branch centers in Nanchang, Hefei, Nanjing, and Xian. Their patients were recruited at either outpatient service or inpatient department and had already made their own choice of moxibustion therapy. Thus, the acupuncture point's Deqi sensation towards manipulation of suspend moxibustion generated the groups to be compared. In trial group, patients felt the Deqi sensation when the acupuncture point was stimulated by moxibustion heat. In the control group, patients only felt local superficial heat sensation (non-Deqi sensation) when the acupuncture point was stimulated by moxibustion heat.

### 2.4. Participants

#### 2.4.1. Recruitment

Patients were recruited in China for this nonrandomized prospective multicentre open comparative cohort study from November 27, 2009, to December 27, 2010. This trial protocol has been approved by local institutional review boards and ethics committees (code issued is 2008(11)) and follows the principles of the Declaration of Helsinki (Edinburgh Version 2000). Oral and written informed consent was obtained after verbal information about the study was provided by the physician.

#### 2.4.2. Inclusion Criteria

Participants were included if they fulfilled the following conditions: (1) participants were diagnosed with LDH according to the guiding principles of clinical research on new drugs [17]; (2) participants were at the age of 18 to 65; participants suffered from moderate to severe LDH, according to the Modified-JOA criteria  (>10 score). Standards of diagnosis were listed as follows: (1) pain occurred in lower back and radiated to the lower limb; (2) limitations of tender point; straight leg raising test and it's strengthen test are positive; (3) skin sensation, muscle strength, and tendon reflex had some changes; (4) changes in spinal posture; (5) X-lateral lumbar spine films showed scoliosis or lumbar lordosis; (6) CT suggestive of disc herniation. Participants were instructed to stop LDH symptomatic relief medication during the run-in and treatment periods and provided the usual care instruction for LDH.

#### 2.4.3. Exclusion Criteria

Patients with any of the following conditions were excluded: (1) patients suffered from serious life-threatening disease, such as the heart disease and disease of brain blood vessels, liver, kidney, or hematopoietic system, and psychotic patients; (2) pregnant women or women in lactation; (3) patients suffered from a single nerve palsy or cauda equina nerve palsy, patients suffered from muscle paralysis or rectum, and Patients presented bladder symptoms; (4) patients complicated with lumbar spinal canal stenosis and space-occupying lesions for other reasons; (5) patients complicated with lumbar spine tumors, infections, tuberculosis, and so forth; (6) patients complicated with moxibustion syncope and unwilling to be treated with moxibustion; (7) patients signed no informed consent.

#### 2.4.4. Interventions and Comparison

Qualified specialists of acupuncture in TCM with at least five years of clinical experience performed the moxibustion in this study. All treatment regimens were standardized between four centers practitioners by means of video, hands-on training, and internet workshops. Both groups of patients were requested to receive no other treatments such as physical therapies, pain-killing medicines, or acupuncture treatment from other places.

In the two groups, 22 millimeter (diameter) × 120 millimeter (length) moxa sticks (made by Jiangxi Provincial TCM Hospital, China) were adopted. The patient usually lied in the comfortable supine position for treatment, with room temperature of 24°C ~ 30°C. Moxibustion was applied simultaneously on the following three acupuncture points: Da Changshu (BL25), Wei Zhong (BL40), and A-Shi Xue (tenderness). The suspended moxibustion was applied 3 centimetre, far from the surface of skin to search for the heat-sensitive moxibustion sensation.

#### 2.4.5. The Heat-Sensitive Moxibustion Sensation Group

In this group, the three acupuncture points were brought mild warmth without burning by moxa sticks and manipulated until the patient reported the characteristic heat sensitization sensation that is commonly called Deqi. Patients felt comfortable in the moxibustion manipulation.

The following patients' sensation suggested the Deqi: penetrating heat sensation due to moxa heat, defined as the heat sensation conducted from the moxa local skin surface into deep tissue, or even into the joint cavity; expanding heat sensation due to moxa heat, defined as the heat sensation spreading the surrounding little by little around the moxa point; transmitting heat sensation due to moxa heat, defined as the heat sensation transferring along some pathway or direction, even to the ankle or hip conduction. In the course of manipulation, the therapist continued for 15 minutes in pertreatment session. Patients received the treatment two times/day in the 1st week (one time/day from the 2nd week) for a total of 18 sessions over 14 days.

#### 2.4.6. The Non-Heat-Sensitive Moxibustion Sensation Group

Common practices were similar to the first group. Only one difference was that patients in this group felt local superficial heat sensation. No Deqi sensations were stimulated in this group.

#### 2.4.7. Outcome Measure

The primary outcome in this trial was measured by Modified-JOA scale. The scale was proposed by Improvement Japanese Orthopaedic Association and was known as the modified edition of JOA Back Pain Evaluation Questionnaire [18]. This scoring system was previously validated [19]. The degree of LDH was divided into three levels: mild: <10, moderate: 10 to 20, and severe: >20. The outcome measured above was assessed before the treatment, 14 days of the last moxibustion session, and 6 months after the last moxibustion session.

#### 2.4.8. Statistical Methods

The statistician was blinded from the allocation of groups. SPSS13.0 and SAS9.0 statistical software packages were used to analyze the data. Statistical analyses were based on the intention-to-treat (ITT) principle, including all patients with baseline values to receive treatment. All tests were exploratory and two-sided with a level of significance of 5%.

We used multilevel models in analysis of covariance (ANCOVA) or generalized estimating equations (GEE). In these models, physicians were considered random effect, and fixed effects were baseline value (continuous), duration of low back pain, patient's age and gender, body mass index (BMI), and Modified-JOA score (continuous). Results are presented as adjusted mean or proportioned with a standard error (SE) and/or 95% confidence interval (CI).

#### 2.4.9. Adverse Events

We defined adverse events as unfavorable or unintended signs; however, symptoms or disease occurred after treatment was not necessarily related to the moxibustion intervention. Adverse events were analyzed descriptively by frequencies, percentages, and Chi-squared or Fisher's exact test (if feasible).

## 3. Results

### 3.1. Population and Baseline

**Figure 1:**
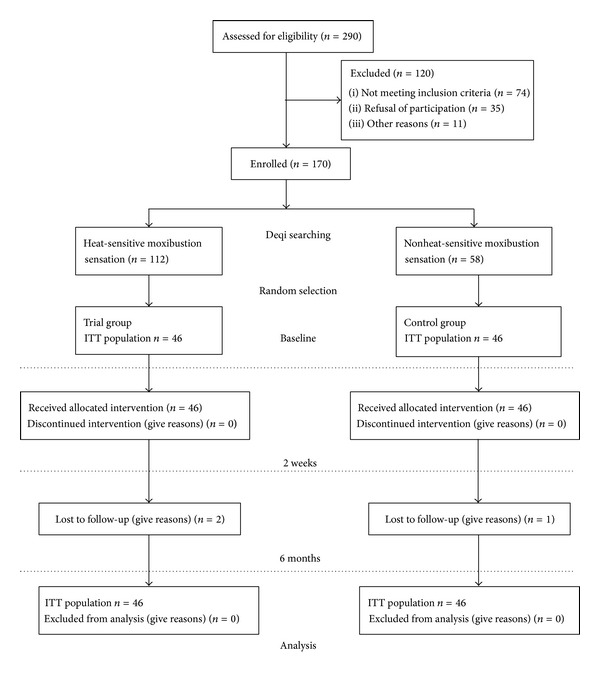
Flow diagram.

Of 290 screened patients, 120 could not be included in the study, mainly because they did not meet all eligibility criteria (Figure 1). After searching for the Deqi, 112 patients experienced heat-sensitive moxibustion sensation; 58 patients had no Deqi sensation. Since a sample of 92 people was calculated in our trial, we selected 46 patients from each queue separately by random drawing.

After six months, data from 89 participants (44 in the trial group and 45 in the control group) were available. Reasons for missing follow-up data included refusal of further participation or being not contactable.

Patient preferences resulted in the following baseline differences: patients in the trial group showed more severe BMI scores, while the Modified-JOA score was higher in the control group. The females of the trial group were more than those of control group. For duration of low back pain, there were obviously differences among the two treatment groups (Table 1). These differences of baseline characteristics were similar to the patients who were still available to be assessed at the 7th month.

### 3.2. Outcome Parameters

After one week, the primary outcome parameter Modified-JOA score showed significant differences between groups: trial group 10.32 ± 4.27 ((adjusted mean ± SE) 95% CI [9.23 ~ 11.40] versus control group 12.42 ± 5.02[11.62 ~ 13.69], *P* = 0.03) (Table 2). Total Modified-JOA score was significantly lower in the trial group in the second week (7.62 ± 4.80[6.46 ~ 8.77 versus 10.56 ± 4.75[9.35 ~ 11.76], *P* = 0.005). Significant differences in total Modified-JOA score were observed between the groups, also evident during the follow-up period (*P* = 0.007).

### 3.3. Safety

No adverse events were reported for the 92 participants.

## 4. Discussions

The comparison of the trial group and control group in this study revealed differences that were statistically significant and clinically relevant in terms of efficacy in reducing Modified-JOA score. The relative change in the mean Modified-JOA score among the heat-sensitive moxibustion sensation group was 10.32 (SE 4.27), compared with 12.42 (SE 5.02) among the non-heat-sensitive moxibustion sensation group in the first week. In the second week, the trend of the differences was enlarged. In addition, significant differences in total Modified-JOA score were observed between the groups, which were also evident during the follow-up period.

To make the most of our knowledge, our study is the largest reported cohort study that compared effectiveness of heat-sensitive moxibustion sensation group with the non-heat-sensitive moxibustion sensation one in the treatment of patients with LDH. Both the evaluation of the results and the statistical analyses were carried out in a blind fashion, in order to improve the objectivity and validity of the study outcomes.

We chose to take the Deqi sensation occurrence preferences into account, making randomization not possible. The groups were homogeneous, according to the baseline evaluation. In the trial group body mass index appeared to be higher compared with the trial group. Conditions according to the Modified-JOA score were less favorable. The gender ratios were different between the two groups. To take baseline differences into account, we adjusted our analyses for these factors. However, it is possible that other unknown and unmeasured factors might have influenced the results. Therefore, the nonrandomized design is a clear limitation of our study considering the internal validity of our results.

In this study, we investigated the relationship between the Deqi sensation and therapeutic effect according to moxibustion stimulation. For acupuncture needle, the needles are inserted into acupuncture points and stimulated until the Deqi is evoked. Multiple unique sensations were experienced by the patients around the applied part, such as suan (aching or soreness), ma (numbness or tingling), zhang (fullness/distention or pressure), and zhong (heaviness) [20]. From a physiological perspective, acupuncture is a modality of sensory stimulation, and the effects obtained are dependent on which sensory receptors are activated, the afferent activity setup, and the resulting activity in the central nervous system [21, 22]. However, the Deqi stimulated by moxibustion is different from the one simulated by acupuncture needle. When the Deqi appears during suspended moxibustion, the patient will have nonlocal or nonsuperficial heat sensation, such as penetrating heat, expanding heat, and transmitting heat. Our trial implemented moxibustion on acupuncture points of Da Changshu (BL25), Wei Zhong (BL40), and A-Shi Xue (tenderness). These acupuncture points were selected because they were commonly used in the treatment of LDH according to the theory of TCM [23–25] Therefore. However, few studies were reported to underlying the mechanisms of Deqi sensation in moxibustion.

The theory of TCM claims that the Deqi is essential to achieve the prospective therapeutic effects. And it was supported by our trial result. The effectiveness of the Deqi sensation might be more superior to the non-Deqi sensation in the treatment of LDH by moxibustion. In a word, it can be inferred that the existence of the Deqi (Qi arrival) phenomenon in the process of suspended moxibustion is closely related to the curative effect, and arrival of heat-sensitive moxibustion sensation could improve the clinical curative effect of moxibustion.

## Figures and Tables

**Table 1:** Baseline characteristics of LDH patients.

Items	Trial group	Control group	*P* value
Age, mean (SD), years	45.65 (10.58)	44.51 (11.46)	0.62
Age > 60 year *n* (%)	12 (26.67%)	14 (31.11%)	0.64
Sex *n* (%)			0.0001
Male	11 (24.44%)	30 (66.67%)	
Female	34 (75.56%)	15 (33.33%)	
Duration of low back pain *n* (%)			0.0001
<1 months	5 (11.11%)	10 (22.22%)	
2–6 months	13 (28.89%)	12 (26.67%)	
7–12 months	6 (27.67%)	12 (26.67%)	
1–5 years	19 (42.22%)	5 (11.11%)	
>5 years	2 (4.44%)	6 (13.33%)	
BMI, mean (SD), kg/m′	25.23 (3.12)	23.24 (3.23)	0.003
Modified-JOA grade *n* (%)			0.21
Severe	30 (66.67%)	24 (53.33%)	
Moderate	15 (33.33%)	21 (46.67%)	
Modified-JOA score at baseline			
Total score mean (SD)	15.23 (4.41)	17.54 (4.57)	0.006

BMI: body mass index; Modified-JOA score: modified Japanese Orthopaedic Association score; SD: standard deviation; LDH: lumbar disc herniation.

**Table 2:** Comparison of modified-JOA scores.

Variable	Week 1		Week 2		Month 6	
	Mean	95% CI	Mean	95% CI	Mean	95% CI
Trial group	10.32	9.23~11.40	7.62	6.46~8.77	6.45	5.62~7.27
Control group	12.42	11.62~13.69	10.56	9.35~11.76	8.36	7.49~9.22
*P* value	0.03		0.005		0.007	

*Adjusted means or proportions and confidence intervals (CI) from multilevel models (ANCOVA or GEE) with fixed effects. All data are intended to treat. In both of the groups, *n* = 46. Modified-JOA score: modified Japanese Orthopaedic Association score; SD: standard deviation; LDH: lumbar disc herniation.
